# Dietary Parsley Seed Mitigates Methomyl-Induced Impaired Growth Performance, Hemato-Immune Suppression, Oxidative Stress, Hepato-Renal Damage, and *Pseudomonas aeruginosa* Susceptibility in *Oreochromis niloticus*

**DOI:** 10.3390/antiox11061185

**Published:** 2022-06-16

**Authors:** Walaa El-Houseiny, Samah Attia Algharib, Eman A. A. Mohamed, Mohamed M. M. Metwally, Yasmina K. Mahmoud, Youssef S. Alghamdi, Mohamed Mohamed Soliman, Yasmina M. Abd-Elhakim, Abd Elhakeem El-Murr

**Affiliations:** 1 Department of Fish Diseases and Management, Faculty of Veterinary Medicine, Zagazig University, Zagazig 44511, Egypt; hakimelmor@zu.edu.eg; 2 Department of Clinical Pathology, Faculty of Veterinary Medicine, Benha University, Moshtohor, Toukh 13736, Egypt; samah.alghareeb@fvtm.bu.edu.eg; 3 National Reference Laboratory of Veterinary Drug Residues (HZAU) and MAO Key Laboratory for Detection of Veterinary Drug Residues, Wuhan 430070, China; 4 Department of Microbiology, Faculty of Veterinary Medicine, Zagazig University, Zagazig 44511, Egypt; eman.zewail@hotmail.com; 5 Department of Pathology, Faculty of Veterinary Medicine, Zagazig University, Zagazig 44511, Egypt; mmetwally@zu.edu.eg; 6 Department of Biochemistry, Faculty of Veterinary Medicine, Suez Canal University, Ismailia 41511, Egypt; yasmina_aziz@vet.suez.edu.eg; 7 Department of Biology, Turabah University College, Taif University, Taif 21995, Saudi Arabia; ysghamdi@tu.edu.sa; 8 Clinical Laboratory Sciences Department, Turabah University College, Taif University, Taif 21995, Saudi Arabia; mmsoliman@tu.edu.sa; 9 Department of Forensic Medicine and Toxicology, Faculty of Veterinary Medicine, Zagazig University, Zagazig 44511, Egypt

**Keywords:** methomyl, parsley seed meal, oxidative stress, *Oreochromis niloticus*, histopathological alterations, disease resistance, immune response, liver, kidney, growth

## Abstract

The present experiment investigated the potential protective role of parsley (*Petroselinum crispum*) seed meal (PSM) in alleviating methomyl (MET)-adverse impacts on growth, whole-body composition, hematological indicators, hepatorenal function, immune response, oxidative status, and disease resistance to *Pseudomonas aeruginosa*. For this purpose, 225 healthy Nile tilapia (*Oreochromis niloticus*) were allotted into five groups (45 fish/group in triplicate). One group was reared in clean water and fed a non-supplemented basal diet, while the other groups were exposed to 20.39 μg L^−1^ MET and fed a non-fortified basal diet or basal diets supplemented with 0.5, 1.0, or 2.0% of PSM for 60 days. The obtained data revealed significantly lower weight gain, feed intake, and specific growth rate, but higher feed conversion ratio and decreases in crude protein, lipid, and ash contents in the MET-exposed fish. Anemia, leukopenia, lymphocytopenia, and esonipenia were also obvious. Furthermore, MET-exposed fish had significantly higher serum levels of hepatic enzymes and renal damage products. Nevertheless, there was a significant depletion of enzymatic and non-enzymatic antioxidants and increased malondialdehyde, myeloperoxidase, and tumor necrosis factor-α levels in MET-exposed fish. The MET exposure significantly depressed lysozyme activity, nitric oxide, complement3, acetylcholinesterase activity, total proteins, globulin, and albumin levels in *O. niloticus* serum. Furthermore, pathological alterations in the liver and kidney were noted. The relative percentage of survival rate in MET-exposed fish was dramatically reduced on day 14 post-challenge with *P. aeruginosa*. The inclusion of PSM, on the other hand, greatly alleviated most of the MET-related negative effects. Taken together, the dietary intervention with PSM has a promising role in alleviating MET-deleterious impacts, rendering parsley seeds a viable aqua feed additive for *O. niloticus*.

## 1. Introduction

Anthropogenic activities and modern lifestyles frequently result in the intentional or unintentional release of thousands of chemicals, including pesticides, into the environment. The aquatic ecosystem is one of the most polluted places, since it is the final resting place for most contaminants [1]. Fish are primarily affected by pesticide hazards due to their absorption through their skin and through gill uptake [2]. Methomyl (MET) is a carbamate pesticide widely used worldwide due to its high efficacy, high solubility, and wide biological effects [3]. Its use has contributed to pest management (controlling insects and nematodes), increasing agricultural productivity. Detectable levels of MET residue have been identified in various water bodies and foods due to its high water solubility, extensive use, and agricultural and industrial release into the environment [4]. MET residue levels in the aquatic environment have been documented to range from 0 to 55.3 g L^−1^ [5].

The World Health Organization (WHO) categorized MET as a very dangerous product (class 1B) [6]. Its exposure poses a serious health risk and causes toxicity and death as it has been found in humans and animals’ blood, liver, kidneys, and brain [7]. Several studies revealed that MET exposure causes serious harm to various biological and metabolic processes in fish organs. For instance, Islamy et al. [8] reported its genotoxic effects on Nile tilapia (*Oreochromis niloticus*). Additionally, Wang Yuqin [9] confirmed the increased oxidative stress in the liver and a decreased growth rate of *O. niloticus* due to MET exposure. Meng et al. [3] recorded the inhibition of the *O. niloticus* antioxidant system when exposed to 0.2–200 μg MET/L^−1^. Moreover, it reduced proinflammatory cytokine expression and abrogated defense against bacterial infections in tilapia [10].

The aquaculture industry must develop nutritional strategies capable of mitigating the risk of waterborne pollution while improving the immunological response and growth of different cultured fish species [11,12,13]. Parsley (*Petroselinum crispum*) is a culinary herb native to the Mediterranean region. Parsley is a member of the Umbelliferae family and is used in the pharmaceutical, cosmetic, and food industries [14]. It contains alpha-linolenic acid, a fatty acid important for growth and reproduction [15]. Flavonoids, terpenoids, carotenoids, myristicin, coumarins, ascorbic acid, and apiole have been identified as major components of parsley [16]. It boosts and promotes organ activity, enhancing their ability to absorb and utilize nutrients [17]. Moreover, it has been shown to have various potential medicinal properties, including antioxidant, antimicrobial, antihyperlipidemic, and hepatoprotective effects [18,19,20]. In modern medicine, parsley has been shown to have a wide range of pharmacological activity, including hepatoprotective, neuroprotective, antidiabetic, analgesic, immunostimulant, antioxidant, anti-platelet, cytoprotective, antibacterial, and antifungal properties [21,22]. However, there is little information about the effects of using PSM as a fish diet supplement.

Nile tilapia (*Oreochromis niloticus*) is a commonly produced fish for human consumption globally [23]. Toxic chemicals in the water, on the other hand, constitute a threat to *O. niloticus* health and growth [24]. To the best of our knowledge, no scientific published study has been conducted on the potential benefits of PSM dietary supplementation in mitigating the MET-induced adverse effects on the health of *O. niloticus*. Consequently, biochemical and histopathological endpoints were assessed, including hematological indices, hepatic enzymes, renal damage products, oxidative stress indices, acetylcholinesterase (AchE) activity, protein profile, and innate immune biomarkers. A comprehensive morphometric histological examination of hepatic and renal tissues in fish exposed to MET and fed a PSM-enriched diet was also carried out. In addition, the fish body’s chemical composition and growth performance were assessed. Moreover, susceptibility to *Pseudomonas aeruginosa* infection, one of the most prevalent bacteria that infect fish and causes significant damage of fish tissues and mortality [25,26], was also investigated.

## 2. Materials and Methods

### 2.1. Tested Compounds and Chemicals

Methomyl (97%) (methyl *N*-(methylcarbamoyloxy) ethanimidothioate was obtained from Sigma Aldrich (St. Louis, MO, USA). Parsley seeds were purchased in dry packages from the local market for herbs and medicinal plants in Zagazig, Egypt. The dried parsley seeds were washed first, dried, and then crushed and kept at 4 °C till use. All other analytical-grade chemicals were purchased from Sigma-Aldrich, St. Louis, MO, USA.

### 2.2. Experimental Fish

Apparently healthy fish, *Oreochromis niloticus*, were gathered from a commercial fish farm in Kafr El-Sheik governorate, Egypt (average body weight: 25.33 ± 0.26 g). Before the experiments, fish were stocked and acclimated for two weeks in 75 L glass aquaria (80 × 40 × 30 cm) with dechlorinated tap water and continual aeration from a central air compressor via an air stone. During the acclimatization period, fish were fed a basal diet. To eliminate excreta, approximately 30% of the water was emptied thrice weekly. The fish were subjected to a photoperiod of 12 h of light and 12 h of darkness. The physicochemical properties of the water used in the aquaria were investigated; the temperature was maintained at 26 ± 0.5 °C, and the pH and dissolved oxygen were kept at 7.5 ± 0.5 and 6.8 ± 0.23 mg L^−1^, respectively. The averages of ammonia, nitrite, and nitrate were recorded at 0.12 ± 0.02, 0.14 ± 0.04, and 2.30 ± 0.05 mg L^−1^, respectively.

The trial was conducted at the Fish Diseases and Management Department, Faculty of Veterinary Medicine, Zagazig University, Egypt. This institution’s Animal Use in Research Committee (IACUC) approved the experiment. The experiments were carried out in compliance with the National Institutes of Health’s (NIH) Ethical Guidelines for the Use and Care of Laboratory Animals in Scientific Investigations.

### 2.3. Diet Formulation and Experimental Plan

Two hundred and twenty-five healthy *O. niloticus* fish were randomly dispersed among five different groups. Each fish group was divided into three aquaria, with 15 fish each. In the control group (G1), fish were fed a basal diet without PSM supplementation and reared in aquaria containing clean water (Table 1), while G2 was exposed to 1/20 LC_50_ (20.39 µg L^−1^) MET [10] and fed a basal diet without PSM supplementation, and G3, G4, and G5 received basal diets enriched with 0.5%, 1.0%, and 2.0% PSM, respectively, with concomitant MET exposure at the same concentration. The experimental diet ingredients were well mixed before being mechanically pelleted, air-dried for 24 h at room temperature, and then stored at 4 °C until use. The basal diet was formulated to meet the optimal dietary needs of fish as specified by the Nutrient Requirements of Fish [27]. Each experimental diet was assessed for crude protein using the macro-Kjeldahl method, crude fat via the ether extraction technique, moisture by a forced-air oven, total ash using a muffle furnace, and crude fiber following AOAC [28] guidelines. Fish were fed until satiation thrice daily (8:00 a.m., 12:00 p.m., and 16:00 p.m.). The trial lasted 60 days. The total fish weight per aquarium was measured every two weeks to monitor fish growth. Every 48 h, the water was fully replaced by moving the fish to aquaria containing freshly prepared MET solutions.

### 2.4. Growth Performance Assessment

To assess growth performance, each replicate’s fish was weighed at the start of the trial and every two weeks. The final body weight (FBW), weight gain (WG), specific growth rate (SGR), weight gain percentage, condition factor (K), feed intake (FI), and feed conversion ratio (FCR) were calculated as follows:

FBW = total weight of fish, divided by the fish number in each replicate
WG = total body weight minus initial body weight
Weight gain percentage = [(final average BW − initial average BW)/initial average BW] × 100
SGR = [(Ln final BW − Ln initial BW)/experiment duration (days)] × 100
FI = feed consumed/number of survival fish
FCR = dry feed fed (g)/wet weight gain (g)
Condition factor (K) = (W/L3) × 100

where W = total fish weight (g) and L = fish length (cm), measured from the tip of the snout to the end of the middle caudal fin.

The mortality rate was calculated using the following formula: mortality rate = (number of dead fish/total initial fish number) × 100.

### 2.5. Whole-Body Chemical Analysis

At the end of the 60-day feeding trial, five fish from each replication were used to analyze the chemical composition of the total fish body. Following AOAC [28] protocols, crude protein levels were determined by a Kjeldahl distillation unit (UDK 129, Velp Scientifica, Usmate Velate, Italy). Moisture was determined by a natural convection oven (JSON-100, Gongju-City, Korea). The ash content was determined using muffle furnaces (Barnstead/Thermolyne Benchtop 47900, Thermo Scientific, Waltham, MA, USA). Soxhlet extractor glassware was also used to estimate crude lipids.

### 2.6. Blood and Tissue Sampling

After the 60-day feeding trial, blood was drawn from caudal vessels and split into two halves. The first half was obtained with EDTA tubes for a complete blood cell count. The second was collected without an anticoagulant until clotting happened; the tubes were kept at 4 °C overnight and centrifuged at 150× *g* for 5 min, followed by 350× *g* for 15 min. The serum was utilized to test immunological function, serum protein electrophoretic pattern, oxidant/antioxidant status, and liver and kidney functions. After collecting blood samples, the fish were dissected to obtain the liver and kidney, then fixed for 48 h in 10% neutral buffered formalin for histopathological investigations.

### 2.7. Estimation of Hematological Values

Hematological indices such as red blood cell counts (RBCs), packed cell volume (PCV), mean corpuscular volume (MCV), hemoglobin content (Hb), mean corpuscular hemoglobin (MCH), and mean corpuscular hemoglobin concentration (MCHC) were determined using a Hema Screen 18 automatic hematology analyzer (Hospitex Diagnostics, Sesto Fiorentino, Italy). Dacie and Lewis’s manual technique was used for counting total leukocytes (WBCs), differential leukocyte counts (lymphocytes, neutrophils, and monocytes), and platelets [29]. 

### 2.8. Serum Biochemical Analysis

#### 2.8.1. Hepatorenal Damage Products

Alanine aminotransferase (ALT), aspartate aminotransferase (AST), lactate dehydrogenase (LDH), alkaline phosphatase (ALP), creatinine, urea, ammonia, total bilirubin, and cholesterol were assessed in serum by Spinreact kits (Esteve De Bas, Girona, Spain) in accordance to the protocols described by Burtis and Ashwood [30], Wenger et al. [31], Murray and Kaplan [32], Pesce [33], Neely and Phillipson [34], Martinen [35], Naito [36], Fossati et al. [37], and Kaplan and Glucose [38], respectively. 

#### 2.8.2. Immune System Response

Serum complement3 (C3) levels were measured by fish-specific ELISA kits and the manufacturer’s instructions. Serum lysozyme activity was determined using spectrophotometry [39]. The concentration of nitric oxide (NO) was determined following the protocol of Dumock [40].

#### 2.8.3. Stress, Inflammatory Status Assays, and Acetyl Choline Esterase Enzyme (AchE) Activity

Stress indicators were measured by commercial colorimetric kits of Biodiagnostic Co. (Cairo, Egypt). The catalase enzyme (CAT) level was determined using the Aebi [41] method. The superoxide dismutase (SOD) activity was measured by the Nishikimi et al. [42] protocol. Malondialdehyde (MDA) was measured according to the Uchiyama and Mihara [43] method. The EnzyChromTM Glutathione Peroxidase Assay Kit (EGPX-100) of Bio-Assay Systems (Hayward, CA, USA) was used to determine quantitative colorimetric glutathione peroxidase (GPx) [44]. 

Myeloperoxidase (MPO) activity in fish serum was determined based on Kumari and Sahoo [45]. Fish ELISA kits of My Biosource Co. for tumor necrosis factor-alpha (TNF-α) were used according to the instructions in the enclosed pamphlets of the kits. The acetylcholinesterase (AchE) activity was evaluated according to Ellman et al. [46]. According to Badawi [47], the electrophoretic pattern of serum proteins, comprising total proteins, albumin, and globulins, was analyzed.

### 2.9. Histopathological Study

At the end of the trial, the fish were necropsied using standard finfish necropsy procedures [48]. Liver and kidney tissue samples were obtained from nine randomly selected fish per group, rinsed in distilled water, and immediately fixed in a 10% neutral buffered formalin solution for 48 h. Post fixation, the specimens were processed using the paraffin technique, sectioned at four μm thick, and stained with hematoxylin and eosin following the protocol described by Suvarna et al. [49]. The stained slides were examined microscopically for morphological alterations. A multiparametric numerical histopathological assessment of the hepatic and renal tissues was performed with minor modifications to the protocol proposed by Bernet et al. [50]. In brief, five non-overlapped randomly chosen microscopic fields (10 objectives) per organ per fish were snapshotted, and the images were then analyzed to calculate lesion frequencies, liver and kidney indices (the higher the indices, the worse the histopathological alterations), and the total indices for a fish using the following formulas:

The following formula determined the frequency of lesions (FQ):

FQ (%) = N_lesion_ × N_total_**^−^**^1^ × 100

where (N_lesion_) represents fish that exhibited a lesion, and (N_total_) represents the total number of fish in the group.

The liver and kidney indices were calculated by the following formula:

Organ index (I_org_) = Σ_rp_ Σ_alt_ (*a*_org rp alt_ × *w*_org rp alt_)

where (rp) is the reaction pattern, (alt) the histopathological alteration, (*a*) the score value (indicates the size of tissue affected by the alteration, and its value ranged between six (diffuse lesion) and zero (lack of the alteration)), and (*w*) the importance factor (indicates how much worse the alteration is, with its value ranging between three and one).

The total index for each individual fish was calculated by adding the indices for the liver and kidney according to the formula:

Total index (Tot-I) = Σ_org_ Σ_rp_ Σ_alt_ (*a*_org rp alt_ × *w*_org rp alt_)

### 2.10. Challenge Test

*Pseudomonas aeruginosa* (previously isolated from naturally infected *O. niloticus* and previously identified and validated as pathogenic) was dispersed on tryptic soy agar (TSA) (Hi-media, Thane West, Maharashtra, India) and incubated for 48 h at 30 °C. The *P. aeruginosa* lethal dose (LD_50_) was evaluated. Fish were intraperitoneally (IP) injected with diverse doses of 24-h live bacteria; then, the infected fish mortality was reported for 3-days post-injection. The LD_50,_ which resulted in 50% fish mortality, was 1 × 10^8^ CFU/mL. Five fish per replicate (*n* = 15 fish/group) were intraperitoneally injected with 0.5 mL of the produced bacterial solution containing 1 × 10^8^ CFU/mL after the feeding trial (60 days). The injected fish were monitored for two weeks to determine the percentage of mortality and post-mortem lesions.

### 2.11. Statistical Analysis

Using SPSS, the data were statistically investigated using one-way Analysis of Variance (ANOVA) (version 16.0, SPSS Inc., Chicago, IL, USA). A Tukey’s multiple comparisons post hoc test was used to compare means between groups, with statistical significance set at *p* ˂ 0.05. The results were displayed as means ± standard error (SE).

## 3. Results

### 3.1. Growth Performance

The growth performance indicators of fish fed graded levels of PSM and exposed to MET for 60 days are presented in Table 2. The acquired results verified that the FW, WG, WG WG%, DWG, SGR, FI, and K were significantly higher (*p* < 0.05) in MET-exposed fish and fed diets fortified with 0.5%, 1%, and 2% PSM (G3, G4, and G5) or basal diet (G1). Conversely, with increased PSM levels, the FCR decreased in fish exposed to MET and the fish fed the basal diet. The best performance was obtained in G4. Fish groups exposed to MET (G2) showed significantly (*p* < 0.05) lower FW, WG, WG%, DWG, SGR, FI, and K values compared with other groups. On the other hand, fish groups exposed to MET attained higher FCR than others.

### 3.2. Mortality Rate

The MET-exposed fish (G2) and those exposed to MET and fed 0.5% PSM (G3) had significantly (*p* < 0.05) higher mortality rates than fish fed the basal diet (G1) and those fed diets supplemented with 1.0% and 2.0% PSM (G4 and G5) (Table 2).

### 3.3. Whole-Body Composition

As shown in Table 2, the analysis of fish body composition revealed significant effects of dietary treatments. The fish fed a basal diet and exposed to MET (G2) displayed lower crude protein, lipids, and ash content than the other experimental groups. The fish exposed to MET and supplemented with PSM revealed significantly higher crude protein and lipid content than the G2 group (*p* < 0.05). The MET-exposed fish fed a supplemented diet with 2.0% PSM (G5) attained higher results than other MET-exposed groups.

### 3.4. Effects on Hematological Indices

The effects of MET exposure and dietary PSM supplementation in the diets of *O. niloticus* fish on hematological indices are displayed in Table 3. The erythrogram revealed that mean values for RBCs, Hb, PCV, and MCHC% were significantly lower in fish fed a basal diet and exposed to MET (G2) than those in control or supplemented groups with PSM. However, supplementing the MET-exposed fish with PSM significantly counteracted the MET-induced anemic condition. Fish that were fed with 1.0% of PSM had the best recovery. Regarding leukograms, fish exposed to MET showed a significant reduction in total WBCs, lymphocytes, eosinophils, heterophils, and monocyte levels compared to values in other groups. On the other hand, PSM supplementation significantly augmented the total WBCs, heterophils, lymphocytes, eosinophils, and monocytes counts relative to those in fish exposed to MET. Groups exposed to MET and supplemented with 1.0% PSM (G4) attained the best results, close to those of the controls.

### 3.5. Blood Biochemical Parameters

#### 3.5.1. Hepatorenal Damage Products

In the current study, MET-exposed fish supplemented with PSM (G3, G4, and G5) showed enhanced liver and kidney function, as demonstrated by the reduced ALT, AST, LDH, urea, creatinine, ammonia, cholesterol, and total bilirubin concentrations relative to those in fish exposed to MET but without supplemented diet (G2). The supplementation of a 1.0% PSM (G4) based diet concomitantly with the MET exposure had the best result, which attained the control values (Table 4).

#### 3.5.2. Immune Response

The experimental treatments significantly influenced the innate immunity parameters (lysozyme, C3, and NO) in the current study (Table 4). The MET-exposed group had significantly lower serum humoral immunity variables such as lysozyme, C3, and NO than the other treatments. In contrast, in the groups fed a PSM-enriched diet and exposed to MET, the earlier immune indicators significantly improved compared to the MET-exposed group. Moreover, a significant enhancement in C3 concentration and lysozyme activity was noted, which attained the control level in the 1.0% PSM (G4) group but was still lower in the 0.5% and 2.0% PSM (G3 and G5) groups. In all PSM supplemented groups, however, the improvement in NO remained significantly lower than that observed in the control group.

#### 3.5.3. Stress, Inflammatory Status Assays, and AChE Activity

Exposure to MET (G2) significantly (*p <* 0.05) decreased the serum concentrations of SOD, CAT, and GPX relative to those of the other fish groups (Table 5). Moreover, it significantly increased MDA, TNF-α, and MPO levels (*p <* 0.05). PSM supplementation in conjunction with MET exposure restored the normal range for these variables but did not achieve the control level. The best results were obtained when a 1.0% PSM (G4) supplement was added to a basal diet. MET-exposed fish had significantly lower serum amounts of total protein, globulin, and albumin than other groups. The addition of PSM alongside MET exposure reduced the decreases in protein profile metrics in all supplemented groups. Yet, the total protein, globulins, and albumin values were still significantly different from controls in groups supplemented with 0.5% and 2.0% PSM (G3 and G5). A significant (*p <* 0.05) decline in serum AChE was obvious in the MET-exposed group (G2), but the dietary addition of PSM significantly (*p <* 0.05) promoted AChE activity, which became not significant (*p <* 0.05) in the control group and groups supplemented with 1.0% and 2.0% PSM (G4 and G5).

### 3.6. Histopathological Findings

#### 3.6.1. Liver

**Figure 1:**
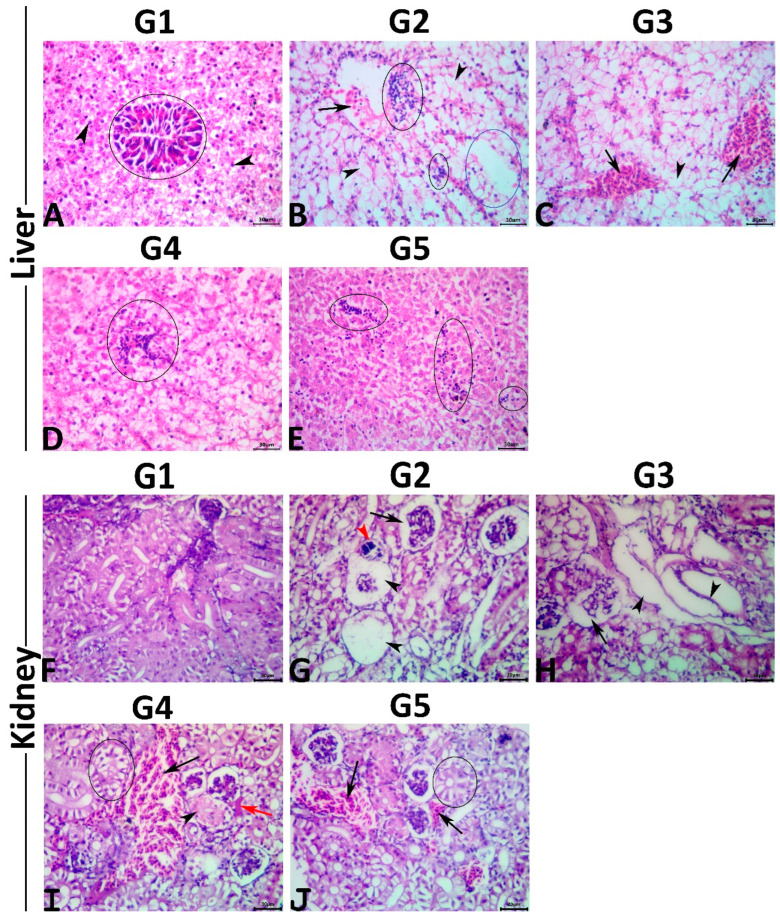
(**A**–**E**); Representative photomicrograph of H&E-stained hepatic tissue sections showing a normal histological picture in the normal control fish (**A**); polyhedral hepatocytes (arrows), exocrine pancreas around portal vein (ellipse). MET-exposed fish showing vascular congestion (arrow), single-cell necrosis (arrowheads), multifocal mononuclear cell aggregations (black ellipse), and vacuolation foci (blue ellipse) (**B**). MET+ 0.5% PSM-treated fish showing vascular congestions (arrows), and single-cell necrosis (arrowhead) (**C**). MET+ 1% PSM-treated fish showing focal mononuclear cell aggregation (ellipse) (**D**). MET+ 0.5% PSM-treated fish showing multifocal minute mononuclear cell infiltrations (ellipses) (**E**). Scale bar 30 μm. (**F**–**J**); Representative photomicrograph of H&E-stained renal tissue sections showing a normal histological picture in the normal control fish (**F**). MET-exposed fish showing glomerular collapse (arrow), glomerular necrosis (black arrowheads), and calcified necrotic tubule (red arrowhead) (**G**). MET+ 0.5% PSM-treated fish showing collapsed glomerulus (arrow), and dilated tubules with flattened epithelial lining (arrowheads) (**H**). MET+ 1% PSM-treated fish showing vascular congestion (black arrow), interstitial edema (red arrow), tubular vacuolation (ellipse), and tubular coagulative necrosis (arrowhead) (**I**). MET+ 0.5% PSM-treated fish showing tubular vacuolation (ellipse), and vascular congestions (arrows) (**J**). Scale bar 30 μm.

The hepatic tissue sections of the control fish revealed normal histological pictures of the hepatopancreas (Figure 1A). Typically, the hepatic parenchyma is surrounded by a very thin fibroconnective capsule and composed primarily of branched two-cell thick cords. These cords are composed of polyhedral hepatocytes with prominent spherical central nuclei containing one nucleolus. The cytoplasm of hepatocytes contained fairly large quantities of glycogen and/or lipid. Hepatic sinusoids were present between these cords and lined with fenestrated endothelium containing elongated nuclei protruding into the sinusoidal lumen. The Von Kupffer cells were not found. The branches of portal veins were surrounded by scattered exocrine acinar pancreatic tissue composed of clusters of pyramidal cells with clear basal nuclei and deep basophilic cytoplasm containing numerous prominent eosinophilic zymogen granules. Exposure to MET induced a diverse range of hepatopathic alterations, including inflammatory (focal and/or diffuse inflammatory cell infiltrate lymphocytes particularly), circulatory (congestions, sinusoidal dilatations, minute hemorrhages, and interstitial edema), regressive (cellular swelling with extensive vacuolations, single-cell necrosis, vacuolation foci, and coagulative necrotic foci), and progressive (focal areas of regenerated hepatocytes, and melanomacrophage aggregate hyperplasia) alterations (Figure 1B). No preneoplastic, dysplastic, or neoplastic alterations were seen in the hepatocytes or the cholangiocytes. Concurrent PSM supplementation with MET induced variable hepatoprotective effects against the MET-induced hepatopathy, as a moderate rescue effect was seen in the hepatic tissue sections of fish fed with 0.5% PSM. In comparison, notable rescue effects were seen in those treated with 1.0% and 2.0% PSM. Concisely, the livers of the fish exposed to MET and treated with 0.5% PSM revealed a significant reduction in the severities but not the frequencies of the circulatory and inflammatory changes associated with a non-significant decline in the retrogressive and progressive alterations. Most specimens of this group exhibited vascular congestions and cytoplasmic vacuolations (Figure 1C). The livers of the fish exposed to MET and treated with 1.0% and 2.0% PSM showed a notable decline in both the severities and frequencies of the MET-induced pathological alterations, yet none of the treatments (1.0% and 2.0%) regained the normal hepatic histology. Most tissue sections in both groups manifested mild degrees of cytoplasmic vacuolations, vascular congestions, and minute lymphocytic aggregations (Figure 1D,E). Multiparametric lesion scoring to the recorded hepatic histological alterations and the hepatic indices in all groups are summarized in Table 6.

#### 3.6.2. Kidneys (Posterior Kidneys)

The renal tissue sections of the control fish showed the normal histological structure of the glomeruli, tubules, and interstitial tissue (Figure 1F). Exposure to MET induced notable nephropathic histological alterations, including inflammatory (interstitial mononuclear cell infiltrations), circulatory (glomerular and interstitial congestions, and interstitial edema), regressive (tubular vacuolations, tubular attenuation, tubular dilatation with flattening of the epithelial lining, single-cell necrosis, focal coagulative necrotic foci, cast formations, glomerular atrophy with widened Bowman’s space, and glomerular necrosis) (Figure 1G), and regressive (tubular regenerations, and melanomacrophage aggregate hyperplasia) alterations with an absence of the neoplastic changes. Concurrent PSM supplementation with MET had average nephroprotective effects. Concisely, the kidneys of the fish exposed to MET and treated with 0.5% PSM revealed a weak rescue effect on the renal histology, as most tissue specimens of this group showed similar but milder lesions to those seen in the MET-exposed fish (Figure 1H). The kidneys of the fish exposed to MET and treated with 1.0% and 2.0% PSM showed significant reductions in both the severities and frequencies of most MET-induced pathological alterations, particularly the retrogressive alterations, yet none of the treatments (1.0% and 2.0%) maintained the normal renal histology. Most tissue sections in both groups showed mild tubular vacuolations, attenuations, glomerular collapse, and vascular congestion (Figure 1I,J). The multiparametric lesion scores for the recorded renal histological alterations and renal indices in all groups are summarized in Table 6.

### 3.7. Effects on Fish Resistance against Challenge with P. aeruginosa 

As presented in Table 7, loss of appetite, loss of reflexes, irregular swimming behavior, and postmortem changes were evident in all groups. Still, they were severe in the fish exposed to MET and fed a basal diet without supplementation (G2). This was also the highest mortality rate among *P. aeruginosa*-challenged fish. After the bacterial challenge, fish fed a basal diet without PSM supplementation and exposed to MET had the lowest RSP. In contrast to the MET-exposed group, all other groups evaluated significantly improved fish resistance to *A. hydrophila* infection. Notably, fish exposed to MET and fed a basal diet containing 1.0% PSM (G4) showed the highest RSP%.

## 4. Discussion

In the present study, MET exposure significantly reduced the *O. niloticus* growth performance in FBW, DWG, SGR, and feed utilization values. These findings followed the study of Mohamed et al. [10], which verified that chronic exposure to MET for 60 days resulted in low growth performance in *O. niloticus*. Additionally, Trachantong et al. [51] reported a decrease in the amphibian growth rate after MET exposure. The MET-induced growth retardation could be related to toxic stress-induced metabolic alterations in protein and carbohydrate metabolism, resulting in energy storage to repair tissue damage, leaving limited energy for growth. Moreover, MET could induce endocrine-disruption, resulting in smaller sizes, as previously reported in exposed tadpoles [51]. Remarkably, concurrent PSM administration in the *O. niloticus* diet improved growth even with MET exposure. It has been demonstrated that spices, herbs, and plant extracts can increase appetite and digestion in fish while also having antibacterial properties [12,52,53]. The PSM growth-augmenting effect could be attributed to the richness of parsley with active compounds (phenols, flavonoids, turbines, and glycosides) that serve as digestive system catalysts by enhancing the elasticity of the intestine’s vasculature [54], thus enhancing the secretion of enzymes (trypsin, chymotrypsin, amylase, lipase) that help in digestive function and excretion [55]. Moreover, the antimicrobial action of parsley is dominated by modulation of the gastrointestinal microbiota [21]. In line with our findings, improved feed conversion rate and growth were demonstrated in *Cyprinus carpio,* which were fed parsley-supplemented diets [56]. Previous studies have shown that parsley has a comparable protective activity against aflatoxins [57] or bifenthrin [58] in *O.* *niloticus*. The mortality of *O. niloticus* was increased by 13.33% after exposure to MET in the current investigation, indicating its significant toxicity. This could be due to MET-induced oxidative stress, immunotoxicity, genotoxicity, and neurobehavioral toxicity, which have previously been documented [10]. Moreover, MET is a potent neurotoxin as it inhibits the AChE enzyme activity, as recorded in the current study. Likely, Jablonski et al. [59] reported that MET affected embryo hatching and larva shape and behavior of zebrafish, resulting in smaller bodies and eyes, failure to inflate the swimming bladder, and hypolocomotor and highly neurotoxic activity. The PSM-enriched feed given concurrently with MET exposure, on the other hand, diminished mortality percent, perhaps via boosting growth performance and immunological response, as well as alleviation of oxidative damage [58]. Moreover, the mitigating effect of parsley was detected on brain neurons, neurotransmitter levels, and neurobehavioral performance in Cd-treated mice [60].

Tissue protein and lipid content are influenced by the dynamic equilibrium between their production and breakdown rates. In this study, the decrease in lipid content and crude protein was evident in the chemical composition analysis of fish exposed to MET. The former observation could be linked to cellular fraction disruption and the resulting impairment in protein synthesis machinery. The intense proteolysis and catabolic effects caused by MET toxicity release free amino acids to meet the increased energy needs caused by toxins [61]. In times of stress, active glycogenolysis and glycolytic pathways offer surplus energy, resulting in decreased crude lipid content [62]. Many studies reported a similar decrease in protein levels and carbohydrate levels [63]. In contrast, PSM dietary supplementation restored crude protein and lipid deposition in the *O. niloticus* muscles at the end of the feeding experiment. This could be due to parsley’s modulatory effect on the activity of fish digestive enzymes and intestinal microbiota, which improves the utilization of digestive products [58]. Increased expression of fatty acid synthesis-related markers and up-regulation of urea cycle components suggest a recovery in oxidative stress and glycolysis [64].

Blood biochemical indicators are crucial for assessing fish health, toxicity, and biomonitoring [65,66,67,68]. Herein, fish exposed to MET developed apparent anemia and a significant decrease in total leukocyte count with a parallel decline in heterophils, lymphocytes, monocytes, and eosinophils. Increased RBCs destruction, osmoregulatory impairment, or decreased erythropoiesis can cause a decline in RBC count [69]. As a compensatory reaction to maintaining gas exchange in damaged gills while decreasing oxygen-carrying capacity, decreases in Hb and PCV values were observed. The decreased leukocyte production could be attributed to depression of leukopoiesis, alteration of the cell membrane or disintegration of white blood cells, and a disruption in the innate immune response [70]. Additionally, lymphocytopenia may be connected to cortisol, a stress hormone that induces lymphocyte death or redistribution from the blood to the tissues [71]. In line with our findings, Mohamed et al. [10] found a decrease in erythrocytic, leukocytic, and Hb content in tilapia fish exposed to MET. MET also affected the erythrogram and total and differential leukocytic counts in rats’ blood [72]. Notably, PSM dietary supplementation significantly reversed the MET-induced hematological alterations. Similarly, parsley extract completely reversed the Hb decrease in *O. niloticus* with aflatoxin contamination [57]. Additionally, the hematological indices of *O. niloticus* fish exposed to MET and treated with taurine showed a significant improvement [10]. This could be due to PSM’s ability to protect the Hb molecule and the erythrocyte membrane from oxidative damage and the resumption of erythropoiesis. The increase in leukocyte and lymphocytic counts may be linked to preserving the leukocyte redox state and the immunostimulant impact of parsley, which boosts leukocyte proliferation.

When a fish is exposed to various water toxicants, its innate immune system, the first line of pathogen defense, is compromised [73]. The innate immune response system relies heavily on various cellular and humoral components [74]. Lysozymes hydrolyze the peptidoglycan layer of the bacterial cell wall by acting as opsonins, causing the complement system and phagocytes to activate [39]. In fish, complement C3 performs various immunological tasks, including removing invading pathogens, initiating inflammatory responses, and clearing other homeostatic cells [75]. NO, which boosts macrophages’ ability to eliminate infections while also playing a role in the inflammatory response, is another key sign of innate immunity [76]. Herein, immunological responses (lysozyme activity, NO, and C3) were significantly reduced in fish exposed to MET, indicating the innate immune response suppression. The decrease in lysozyme after MET exposure in fish could be attributed to decreased leukocyte count and function, including neutrophils and macrophages [77]. Moreover, the noted reduction in NO amount may reveal declined phagocytosis. The immunosuppressive impact of MET could be due to a toxic insult that lowers total leukocytic and lymphocytic counts. It could also result from uncontrolled inflammatory cytokine production and increased lymphoid organ and immune cell apoptosis. Furthermore, MET has been implicated as an endocrine disruptor [51]. In agreement with our findings, the activity of serum lysozyme, C3, and NO was reduced in *O. niloticus* after 60 days of exposure to MET [10]. 

Serum protein, albumin, and globulin levels are also linked to innate immunity [78]. Following 60 days of exposure to MET in *O. niloticus*, a significant fall in total serum protein, albumin, and globulin was detected, indicating immunoglobulin underproduction [79]. Hypoproteinemia was triggered due to catabolism of protein and/or malfunction of the liver [80]. As a result, the depleted immune components and the hypoproteinemic condition were restored when PSM was supplemented. This immunostimulant effect corresponds to an improvement in total leukocytic and lymphocytic counts. Previous research has shown that parsley boosts humoral and cellular immunity [81,82].

During normal metabolism, reactive oxygen species (ROS) such as superoxide anions and hydroxyl radicals are generated in aerobic cells, mostly through mitochondrial oxidative metabolism, and some of these intermediates are thought to be harmful to cells, causing oxidative stress and oxidative damage [83]. Nonetheless, aerobic organisms have evolved mechanisms to activate antioxidant defense systems in different organs and tissues, which include antioxidant enzymes like CAT, SOD, GR, GPx, and GST, as well as antioxidant scavengers to protect against damage caused by high levels of ROS [84]. In the current study, noticeable exhaustion of the GPx, CAT, and SOD activities but increased inflammatory markers (MPO and TNF-*α*) and MDA concentrations were recorded in fish exposed to MET. It is possible to hypothesize that under conditions of chronic exposure to MET, it could not be metabolized and was not entirely eliminated from the tilapia body by the cells. As a result, the MET accumulated in the body and destroyed the protein structures of the antioxidant enzymes, resulting in decreased antioxidant enzyme activity, according to Jagetia and Aggarwal [85]. The conjugation of MET or its degradation products to polyunsaturated fatty acids may be blamed for the MET-induced elevation in MDA levels [86]. In this regard, Mohamed et al. [10] proved that the main toxic effects of MET in *O. niloticus* take place via oxidative stress, resulting in oxidative damage through reducing antioxidant enzyme activity and inducing DNA damage and lipid peroxidation. As proven by the fact that MET can cause oxidative damage, LPO levels are raised, and several antioxidant enzymes are disrupted [87]. In contrast, PSM significantly suppresses the oxidative and lipid peroxidative damage induced by MET by stimulating the production of antioxidant enzymes and thereby boosting cellular antioxidant defenses. Parsley is abundant in polyphenolic flavonoid anti-oxidants such as apiin, apigenin, and luteolin [88]. Along with luteolin and vitamin C, parsley acts as an anti-inflammatory agent, enhancing catalase and glutathione activity and lowering lipid peroxidation [89]. Previous reports have evidenced parsley efficiency in heavy metal excretion from the body [60,90].

The liver is the first organ to be exposed to any foreign molecule delivered by portal circulation, and it sustains the most damage. The release of intracellular enzymes such as AST, ALT, LDH, and ALP into the circulation is one of the most sensitive and dramatic indications of hepatocyte injury in response to toxicity [12,91]. The current findings revealed a significant increase in the activities of AST, ALT, LDH, and ALP in the serum of fish together with metabolic products such as cholesterol and bilirubin, implying that MET could cause liver damage. This changed biochemical profile was linked to a variety of hepatic lesions, including lymphocytic infiltration, coagulative necrotic foci, congestion, sinusoidal dilatations, and melanomacrophage aggregate hyperplasia. Changes in membrane permeability and loss of functional integrity of cell membranes in the liver also cause cellular leakage, resulting in the pervasive release of these enzymes from the cell [92]. Furthermore, MET-induced hypercholesterolemia may be due to organisms’ requirements for additional energy reserves to cope with stressors’ effects [93]. Our observed increase in these parameters due to MET intoxication is in accordance with the findings of Hashish and Elgaml [94]. Restoration of the disturbed enzyme activities and hepatic architecture with the correction of metabolic product concentrations after PSM supplementation is the primary explanation for their hepatoprotective effects. Indeed, the hepatoprotective effect of parsley arises from its capacity to scavenge ROS, as reported in numerous assays [95,96]. The decreased LDH and hypocholesterolemia due to the concurrent treatment with 20% parsley seed methanol extract was documented in rats [97]. The stabilization of serum bilirubin levels by PSM is a clear indication of the improvement in the functional status of the liver cells. This activity could be attributed to the phytochemical analysis’s bioactive components, including flavonoids, lignans, alkaloids, bisbenzyl, coumarins, and terpenes [98].

Fish kidneys gather the most post-branchial blood, so renal diseases could be effective indicators of environmental contamination [99]. MET caused a considerable increase in serum ammonia, urea, and creatinine activity in the present study. The elevated levels of ammonia, urea, and creatinine may be linked to the effects of MET on muscle metabolism and its limited impact on purine metabolism and renal tubule injury. These findings matched those of Hashish and Elgaml [94]. Conversely, the present study found that PSM supplementation might reverse MET-induced nephrotoxicity in *O. niloticus* fish, as indicated by restoration of the disturbed metabolites and renal architecture. Apigenin and its glucosidal flavonoids, which are abundant in parsley, have been discovered to have anti-inflammatory, antioxidant, and anticancer properties, particularly in the case of renal inflammation [100]. A similar nephroprotective effect of PSM has been previously recognized [101].

In the current study, *O. niloticus* was challenged with *P. aeruginosa* after MET exposure for 60 days. The findings demonstrated that the RPS was dropped noticeably in MET-exposed and fed basal diet following the challenge. Moreover, severe abnormal behavior and clinical signs were observed. The loss of cellular and humoral immune components caused by MET could be a major contributor to lowering the RPS and, as a result, compromising the fish response to *P. aeruginosa* in the challenge. Importantly, when PSM was added to the fish diet with MET exposure, it improved the outcomes of suppressed innate immunity against *P. aeruginosa*, which could be strongly linked to the immune-stimulatory activity observed in this trial. Correspondingly, Farag et al. [58] demonstrated that dietary parsley essential oil supplementation in the *O. niloticus* diet alleviated the adverse effects of bifenthrin and enhanced disease resistance against *Aeromonas hydrophila*.

Importantly, the effects of new feed ingredients or additives on fish health indicators and safety should be carefully considered for an appropriate assessment of any nutritional strategy [13]. In this regard, the liver and kidney function indicators are important determinants in assessing feed ingredient safety in fish diets because of their roles in detoxification, biotransformation, and excretion of xenobiotics [102,103]. Of note, in the current study, supplementing fish with diets fortified with up to 2% of PSM, even with MET exposure, did not negatively impacted the liver and kidney function indicators, indicating a wide safety margin of PSM as a feed supplement in fish.

## 5. Conclusions

Finally, the current study verified for the first time that dietary supplementation of PSM can positively affect health against MET-induced growth retardation, anemia, leukopenia, hepato-renal damage, immunosuppression, AchE inhibition, and weakened disease resistance in *O. niloticus*. The antioxidant and anti-inflammatory activities could be the probable underlying mechanisms of PSM. Additionally, it has the potential to recover the histopathological architecture of the liver and kidney. The prevailing study’s findings may point to PSM as an eco-friendly aqua-feed supplement that enhances fish health even with waterborne contaminants. Future mechanistic studies are needed to elucidate PSM’s other probable underlying mechanisms of action, focusing on the genome transcriptome and proteome. Moreover, further studies assessing the effects of PSM on MET accumulation in fish tissues after different periods of exposure and recoveries are required.

## Figures and Tables

**Table 1:** Ingredients and chemical compositions of the experimental diets.

Ingredients	Experimental Diets (%)			
	Control	0.5%	1.0%	2.0%
Fish meal, 60%	15	15	15	15
Poultry by-product meal, 60%	11	11	11	11
Soybean meal, 46.5%	28	28	28	27
Yellow corn	31	30	30	30
Wheat flour	8	8.5	8	8
Parsley seeds (PS)	0	0.5	1	2
Vegetable oil	4	4	4	4
Vitamin premix ^1^	1.5	1.5	1.5	1.5
Mineral premix ^2^	1.5	1.5	1.5	1.5
Total	100	100	100	100
Analyzed composition (%) as fed basis				
Crude protein (N × 6.25)	31.29	30.98	31.16	31.25
Crude lipid	9.38	10.25	9.90	9.80
Crude fiber	2.57	3.88	3.11	3.10
Ash	7.10	7.50	7.65	7.25
Nitrogen free extract (NFE) ^3^	49.66	47.39	48.18	48.60
DE, kcal/kg diet ^4^	3096.43	3099.30	3097	3102.55

^1^ Vitamin premix (per kg of premix): vitamin A, 8,000,000 IU; vitamin E, 7000 mg; vitamin D3, 2,000,000 IU; vitamin K3, 1500 mg; biotin, 50 mg; folic acid, 700 mg; nicotinic, 20,000 mg; pantothenic acid, 7000 mg; vitamin B1, 700 mg; vitamin B2, 3500 mg; vitamin B6, 1000 mg; vitamin B12, 7 mg. ^2^ Mineral premix (per kg of premix): zinc sulfate, 4.0 g; iron sulfate, 20 g; manganese sulfate, 5.3 g; copper sulfate, 2.7 g; calcium iodine, 0.34 g; sodium selenite, 70 mg; cobalt sulfate, 70 mg; and CaHPO_4_·2H_2_O up to 1 kg. ^3^ Nitrogen free extract (NFE) calculated by difference (100 − protein% + lipids% + ash% + crude fiber%). ^4^ Digestible energy (DE) calculation based on protein values 3.5 kcal/g, fat 8.1 kcal/g, NFE 2.5 kcal/g.

**Table 2:** Effect of parsley seed meal (PSM) supplementation on growth performance and whole body composition (% fresh weight basis) of *O. niloticus* exposed to methomyl (MET) 20.39 µg/L for 60 days.

	Experimental Groups				
	G1	G2	G3	G4	G5
**Growth indices**					
Initial body weight (g)	25.33 ^a^ ± 0.26	25.13 ^a^ ± 0.26	24.96 ^a^ ± 0.32	25.23 ^a^ ± 0.29	25.16 ^a^ ± 0.53
Final body weight (g)	55.53 ^a^ ± 0.37	41.00 ^c^ ± 0.57	49.80 ^b^ ± 0.43	54.60 ^a^ ± 0.43	53.50 ^a^ ± 0.32
Weight gain (g)	30.20 ^a^ ± 0.11	15.86 ^c^ ± 0.48	24.83 ^b^ ± 0.72	29.36 ^a^ ± 0.38	28.33 ^a^ ± 0.27
Weight gain %	119.22 ^a^ ± 0.77	63.13 ^c^ ± 1.96	99.56 ^b^ ± 4.08	116.41 ^a^ ± 2.18	112.72 ^a^ ± 3.33
Daily weight gain (g)	0.50 ^a^ ± 0.001	0.26 ^c^ ± 0.008	0.41 ^b^ ± 0.01	0.48 ^a^ ± 0.006	0.47 ^a^ ± 0.004
Specific growth rate (%)	0.56 ^a^ ± 0.002	0.35 ^c^ ± 0.008	0.49 ^b^ ± 0.01	0.55 ^a^ ± 0.007	0.54 ^a^ ± 0.01
Feed intake (g)	48.00 ^a^ ± 01.15	33.33 ^c^ ± 0.88	43.66 ^b^ ± 0.57	49.00 ^a^ ± 0.57	0.47 ^a^ ± 0.004
Feed conversion ratio	1.58 ^c^ ± 0.03	2.09 ^a^ ± 0.03	1.73 ^b^ ± 0.03	1.67 ^bc^ ± 0.02	1.65 ^bc^ ± 0.01
Condition factor (K)	1.64 ^a^ ± 0.02	1.12 ^c^ ± 0.009	1.44 ^b^ ± 0.02	1.61 ^a^ ± 0.03	1.61 ^a^ ± 0.03
Mortality (%)	2.22 ^b^ ± 2.22	13.33 ^a^ ± 0.00	6.66 ^ab^ ± 0.00	2.22 ^b^ ± 2.22	4.42 ^b^ ± 2.21
**Whole body composition (%)**					
Moisture	75.33 ^ab^ ± 0.60	74.43 ^bc^ ± 0.31	73.83 ^c^ ± 0.12	75.40 ^ab^ ± 0.51	76.06 ^a^ ± 0.46
Crude lipids	10.53 ^a^ ± 0.49	4.73 ^d^ ± 0.21	7.50 ^c^ ± 0.28	9.10 ^b^ ± 0.37	10.20 ^ab^ ± 0.47
Crude protein	48.33 ^a^ ± 0.60	39.13 ^d^ ± 0.40	44.86 ^c^ ± 0.26	46.96 ^b^ ± 0.26	47.33 ^ab^ ± 0.37
Ash	12.36 ^a^ ± 0.29	8.63 ^b^ ± 0.46	9.73 ^b^ ± 0.29	11.23 ^a^ ± 0.40	11.10 ^a^ ± 0.43

Values are represented as the mean ± SE, *n* = 3 replicates (15 fish/replicate). The means within the same row carrying different superscripts (^a–d^) are significant at *p* < 0.05. G1, negative control, was fed a basal diet. G2 was exposed to MET 20.39 µg/L and fed a basal diet without PSM. G3 was exposed to MET 20.39 µg/L and fed a basal diet with 0.5% of PSM. G4 was exposed to MET 20.39 µg/L and fed a basal diet with 1.0% of PSM. G5 was exposed to MET 20.39 µg/L and fed a basal diet with 2.0% of PSM.

**Table 3:** Effect of parsley seed meal (PSM) supplementation on hematological indices of *O. niloticus* exposed to methomyl (MET) 20.39 µg/L for 60 days.

	Experimental Groups				
	G1	G2	G3	G4	G5
**Erythrogram**					
RBCs (10^6^/mm^3^)	2.78 ^b^ ± 0.008	1.18 ^d^ ± 0.008	1.75 ^c^ ± 0.01	2.85 ^a^ ± 0.01	2.76 ^b^ ± 0.02
Hb (gm/dL)	9.20 ^a^ ± 0.05	3.25 ^d^ ± 0.01	5.50 ^c^ ± 0.05	9.33 ^a^ ± 0.04	8.82 ^b^ ± 0.04
PCV (%)	30.30 ^a^ ± 0.05	17.33 ^d^ ± 0.17	23.50 ^c^ ± 0.28	30.79 ^a^ ± 0.02	29.66 ^b^ ± 0.27
MCV (fL)	108.73 ^c^ ± 0.50	146.09 ^a^ ± 2.52	134.32 ^b^ ± 2.56	108.04 ^c^ ± 0.35	107.48 ^c^ ± 0.47
MCHC (%)	30.36 ^a^ ± 0.16	18.74 ^c^ ± 0.09	23.40 ^b^ ± 0.27	30.30 ^a^ ± 0.11	29.73 ^a^ ± 0.36
**Leukogram**					
WBCs (10^3^/mm^3^)	5.26 ^a^ ± 0.03	2.35 ^d^ ± 0.08	4.15 ^c^ ± 0.04	5.14 ^ab^ ± 0.03	5.00 ^b^ ± 0.02
Lymphocytes (10^3^/mm^3^)	2.91 ^a^ ± 0.01	1.13 ^d^ ± 0.008	2.42 ^c^ ± 0.01	2.86 ^ab^ ± 0.01	2.81 ^b^ ± 0.01
Heterophils (10^3^/mm^3^)	1.38 ^a^ ± 0.01	0.80 ^c^ ± 0.05	1.13 ^b^ ± 0.01	1.35 ^a^ ± 0.008	1.32 ^a^ ± 0.005
Eosinophils (10^3^/mm^3^)	0.33 ^a^ ± 0.005	0.14 ^d^ ± 0.005	0.20 ^c^ ± 0.01	0.30 ^ab^ ± 0.005	0.27 ^b^ ± 0.005
Monocytes (10^3^/mm^3^)	0.64 ^a^ ± 0.005	0.28 ^d^ ± 0.01	0.40 ^c^ ± 0.01	0.63 ^ab^ ± 0.005	0.60 ^b^ ± 0.005

Values are represented as the mean ± SE, *n* = 3 replicates (5 fish/replicate). The means within the same row carrying different superscripts (^a–d^) are significant at *p* < 0.05. G1, negative control, was fed a basal diet. G2 was exposed to MET 20.39 µg/L and fed a basal diet without PSM. G3 was exposed to MET 20.39 µg/L and fed a basal diet with 0.5% of PSM. G4 was exposed to MET 20.39 µg/L and fed a basal diet with 1.0% of PSM. G5 was exposed to MET 20.39 µg/L and fed a basal diet with 2.0% of PSM. RBCs: red blood cells; Hb: hemoglobin; PCV: packed cell volume; MCV: mean corpuscular volume; MCH: mean corpuscular hemoglobin; MCHC: mean corpuscular hemoglobin concentration; WBC: white blood cells.

**Table 4:** Immunological response as well as liver and kidney function tests of *O. niloticus* exposed to methomyl (MET) 20.39 µg/L and fed parsley seed meal (PSM) for 60 days.

Experimental Groups					
	G1	G2	G3	G4	G5
**Lysozyme (µg/mL)**	19.36 ^b^ ± 0.41	7.23 ^e^ ± 0.14	13.56 ^d^ ± 0.15	20.11 ^a^ ± 0.15	18.34 ^c^ ± 0.08
**Complement3 (ug/mL)**	107.78 ^a^ ± 0.77	62.56 ^d^ ± 1.08	86.26 ^c^ ± 0.67	106.38 ^ab^ ± 0.59	104.72 ^b^ ± 0.98
**NO (µmol/L)**	49.16 ^a^ ± 0.60	19.53 ^e^ ± 0.43	36.13 ^d^ ± 0.49	47.60 ^b^ ± 0.40	45.63 ^c^ ± 0.40
**ALT (U/L)**	13.24 ^c^ ± 0.43	67.83 ^a^ ± 1.30	37.46 ^b^ ± 1.67	15.23 ^c^ ± 0.26	16.40 ^c^ ± 0.28
**AST (U/L)**	26.40 ^c^ ± 0.32	54.61 ^a^ ± 2.27	39.02 ^b^ ± 0.73	28.50 ^c^ ± 0.34	29.50 ^c^ ± 0.34
**LDH (U/L)**	1491.91 ^c^ ± 1.96	1883.56 ^a^ ± 8.77	1609.63 ^b^ ± 2.22	1498.71 ^c^ ± 0.77	1501.33 ^c^ ± 1.86
**ALP (IU/L)**	24.41 ^b^ ± 0.51	17.23 ^c^ ± 0.54	24.16 ^b^ ± 0.65	26.20 ^a^ ± 0.49	27.58 ^a^ ± 0.32
**Ammonia (µg/dL)**	226.33 ^d^ ± 0.95	313.70 ^a^ ± 0.25	274.43 ^b^ ± 1.85	229.56 ^c^ ± 0.35	230.46 ^c^ ± 0.31
**Total bilirubin (mg/dL)**	0.28 ^d^ ± 0.005	0.97 ^a^ ± 0.008	0.62 ^b^ ± 0.011	0.30 ^cd^ ± 0.005	0.31 ^c^ ± 0.005
**Cholesterol (mg/dL)**	194.30 ^d^ ± 1.04	285.33 ^a^ ± 0.78	245.50 ^b^ ± 1.10	198.16 ^c^ ± 0.60	199.63 ^c^ ± 0.37
**Urea (mg/dL)**	2.81 ^d^ ± 0.008	9.50 ^a^ ± 0.06	5.30 ^b^ ± 0.02	2.87 ^cd^ ± 0.01	2.92 ^c^ ± 0.01
**Creatinine (mg/dL)**	0.42 ^d^ ± 0.008	0.94 ^a^ ± 0.01	0.55 ^b^ ± 0.01	0.45 ^d^ ± 0.005	0.48 ^c^ ± 0.008

Values are represented as the mean ± SE, *n* = 3 replicates (5 fish/replicate). The means within the same row carrying different superscripts (^a–e^) are significant at *p* < 0.05. G1, negative control, was fed a basal diet. G2 was exposed to MET 20.39 µg/L and fed a basal diet without PSM. G3 was exposed to MET 20.39 µg/L and fed a basal diet with 0.5% of PSM. G4 was exposed to MET 20.39 µg/L and fed a basal diet with 1.0% of PSM. G5 was exposed to MET 20.39 µg/L and fed a basal diet with 2.0% of PSM. NO: nitric oxide. ALT: alanine aminotransferase; AST: aspartate transaminase; LDH: lactate dehydrogenase; ALP: alkaline phosphatase.

**Table 5:** Oxidative, inflammatory biomarkers, acetyl cholinesterase (AchE) activity, and protein profile of *O. niloticus* exposed to methomyl (MET) (20.39 µg/L) and fed parsley seed meal (PSM) for 60 days.

Experimental Groups					
	G1	G2	G3	G4	G5
**MDA (nmol/mL)**	12.66 ^d^ ± 0.48	36.43 ^a^ ± 0.48	21.36 ^b^ ± 0.58	14.33 ^c^ ± 0.49	14.76 ^c^ ± 0.29
**SOD (U/mL)**	5.23 ^a^ ± 0.35	0.80 ^d^ ± 0.05	2.46 ^c^ ± 0.23	4.43 ^b^ ± 0.24	3.83 ^b^ ± 0.17
**GPX (U/L)**	156.00 ^a^ ± 1.73	106.00 ^d^ ± 0.57	133.00 ^c^ ± 1.15	153.00 ^ab^ ± 0.57	150.00 ^b^ ± 1.15
**CAT (U/L)**	76.10 ^a^ ± 0.46	48.16 ^e^ ± 0.40	59.50 ^d^ ± 0.34	73.83 ^b^ ± 0.40	71.90 ^c^ ± 0.46
**TNF-α (pg/mL)**	15.76 ^d^ ± 0.24	42.93 ^a^ ± 0.26	23.86 ^b^ ± 0.20	17.03 ^c^ ± 0.46	17.66 ^c^ ± 0.40
**MPO (U/L)**	73.33 ^e^ ± 0.49	118.40 ^a^ ± 0.49	94.36 ^b^ ± 0.46	76.50 ^d^ ± 0.34	78.36 ^c^ ± 0.34
**AchE (U/L)**	504.76 ^a^ ± 1.21	322.86 ^c^ ± 1.33	475.33 ^b^ ± 1.47	500.03 ^a^ ± 1.29	499.13 ^a^ ± 1.32
**Total proteins (g/dL)**	5.80 ^a^ ± 0.05	2.60 ^d^ ± 0.17	4.16 ^c^ ± 0.17	6.00 ^a^ ± 0.05	5.08 ^b^ ± 0.09
**Albumin (g/dL)**	2.35 ^b^ ± 0.07	1.00 ^d^ ± 0.05	1.70 ^c^ ± 0.17	2.92 ^a^ ± 0.13	2.14 ^b^ ± 0.03
**Total globulins (g/dL)**	3.45 ^a^ ± 0.10	1.60 ^d^ ± 0.11	2.46 ^c^ ± 0.033	3.08 ^b^ ± 0.08	2.94 ^b^ ± 0.12

Values are represented as the mean ± SE, *n* = 3 replicates (5 fish/replicate). The means within the same row carrying different superscripts (^a–e^) are significant at *p* < 0.05. G1, negative control, was fed a basal diet. G2 was exposed to MET 20.39 µg/L and fed a basal diet without PSM. G3 was exposed to MET 20.39 µg/L and fed a basal diet with 0.5% of PSM. G4 was exposed to MET 20.39 µg/L and fed a basal diet with 1.0% of PSM. G5 was exposed to MET 20.39 µg/L and fed a basal diet with 2.0% of PSM. MDA: malondialdehyde; SOD: super oxide dismutase; GPx: glutathione peroxidase; CAT: catalase; TNF-α: tumor necrosis factor-alpha; MPO: myeloperoxidase. AchE: acetyl cholinesterase.

**Table 6:** Effect of parsley seed meal (PSM) supplementation on lesion scores of liver and kidney tissues of *O. niloticus* exposed to methomyl (MET) (20.39 µg/L) for 60 days.

Organ	Histopathological Alteration			G1		G2		G3		G4		G5	
	Reaction Pattern	Type	IF	FQ (%)	Index	FQ (%)	Index	FQ (%)	Index	FQ (%)	Index	FQ (%)	Index
**Liver**	Inflammatory alterations	Leukocytic infiltration	2	0	0.00 ^b^ ± 0.00	80	1.60 ^a^ ± 0.26	40	0.80 ^ab^ ± 0.32	40	0.80 ^ab^ ± 0.32	40	0.80 ^ab^ ± 0.32
	Circulatory alterations	Congestion	1	0	0.00 ^b^ ± 0.00	80	1.80 ^a^ ± 0.41	60	0.70 ^b^ ± 0.21	40	0.40 ^b^ ± 0.16	30	0.30 ^b^ ± 0.15
		Edema	1	0	0.00^b^ ± 0.00	60	0.70^a^ ± 0.21	30	0.30^ab^ ± 0.15	20	0.20^ab^ ± 0.13	20	0.20^ab^ ± 0.13
		Hemorrhages	1	0	0.00^a^ ± 0.00	20	0.20^a^ ± 0.13	10	0.10^a^ ± 0.10	0	0.00^a^ ± 0.00	0	0.00^a^ ± 0.00
	Regressive alterations	Cellular swelling	1	0	0.00 ^c^ ± 0.00	100	3.00 ^a^ ± 0.39	80	1.30 ^b^ ± 0.30	60	0.90 ^bc^ ± 0.23	50	0.80 ^bc^ ± 0.29
		Cytoplasmic vacuolations	1	0	0.00 ^c^ ± 0.00	100	1.80 ^a^ ± 0.32	90	1.20 ^ab^ ± 0.20	80	0.90 ^b^ ± 0.17	80	0.80 ^b^ ± 0.13
		Vacuolation foci	2	0	0.00 ^a^ ± 0.00	30	0.60 ^a^ ± 0.30	10	0.20 ^a^ ± 0.20	0	0.00 ^a^ ± 0.00	0	0.00 ^a^ ± 0.00
		Single-cell necrosis	3	0	0.50 ^b^ ± 0.16	100	3.80 ^a^ ± 0.41	60	1.80 ^b^ ± 0.48	30	0.90 ^b^ ± 0.45	30	0.90 ^b^ ± 0.45
		Coagulative necrotic foci	3	0	0.00 ^a^ ± 0.00	30	0.90 ^a^ ± 0.45	20	0.60 ^a^ ± 0.40	0	0.00 ^a^ ± 0.00	0	0.00 ^a^ ± 0.00
	Progressive alterations	Regenerated hepatocytes	2	0	0.00 ^a^ ± 0.00	30	0.60 ^a^ ± 0.30	30	0.60 ^a^ ± 0.30	40	0.80 ^a^ ± 0.32	40	0.80 ^a^ ± 0.32
		Melanomacrophage hyperplasia	2	0	0.00 ^a^ ± 0.00	20	0.40 ^a^ ± 0.26	20	0.40 ^a^ ± 0.26	30	0.60 ^a^ ± 0.30	20	0.40 ^a^ ± 0.26
**I_L_**			NA	NA	0.50 ^c^ ± 0.16	NA	15.40 ^a^ ± 1.63	NA	8.00 ^b^ ± 2.04	NA	5.20 ^bc^ ± 1.18	NA	5.00 ^bc^ ± 1.29
**Kidney**	Inflammatory alterations	Leukocytic infiltration	2	0	0.00 ^a^ ± 0.00	40	0.80 ^a^ ± 0.32	30	0.60 ^a^ ± 0.30	20	0.40 ^a^ ± 0.26	10	0.20 ^a^ ± 0.20
	Circulatory alterations	Congestion	1	0	0.00 ^b^ ± 0.00	80	1.20 ^a^ ± 0.29	50	0.60 ^ab^ ± 0.22	30	0.30 ^b^ ± 0.15	40	0.40 ^b^ ± 0.16
		Edema	1	0	0.00 ^a^ ± 0.00	30	0.30 ^a^ ± 0.15	30	0.30 ^a^ ± 0.15	10	0.10 ^a^ ± 0.10	20	0.20 ^a^ ± 0.13
		Hemorrhages	1	0	0.00 ^a^ ± 0.00	20	0.20 ^a^ ± 0.13	10	0.10 ^a^ ± 0.10	10	0.10 ^a^ ± 0.10	10	0.10 ^a^ ± 0.10
	Regressive alterations	Glomerular collapse	2	0	0.00 ^a^ ± 0.00	40	0.80 ^a^ ± 0.32	30	0.60 ^a^ ± 0.30	10	0.20 ^a^ ± 0.20	10	0.20 ^a^ ± 0.20
		Glomerular necrosis	3	0	0.00 ^a^ ± 0.00	40	1.00 ^a^ ± 0.44	20	0.40 ^a^ ± 0.26	10	0.20 ^a^ ± 0.20	10	0.20 ^a^ ± 0.20
		Tubular vacuolation	1	0	0.00 ^c^ ± 0.00	100	2.70 ^a^ ± 0.57	90	1.30 ^b^ ± 0.26	40	0.40 ^bc^ ± 0.16	50	0.50 ^bc^ ± 0.16
		Tubular necrosis	3	0	0.50 ^a^ ± 0.16	60	1.20 ^a^ ± 0.66	40	1.20 ^a^ ± 0.48	10	0.30 ^a^ ± 0.30	10	0.30 ^a^ ± 0.30
		Tubular dilatation	2	0	0.00 ^a^ ± 0.00	20	0.40 ^a^ ± 0.26	20	0.40 ^a^ ± 0.26	0	0.00 ^a^ ± 0.00	0	0.00 ^a^ ± 0.00
		Cast formation	1	0	0.00 ^b^ ± 0.00	40	0.70 ^a^ ± 0.33	30	0.30 ^ab^ ± 0.15	0	0.00 ^b^ ± 0.00	10	0.10 ^ab^ ± 0.10
	Progressive alterations	Regenerated tubular epithelium	2	0	0.00 ^a^ ± 0.00	30	0.60 ^a^ ± 0.30	20	0.40 ^a^ ± 0.26	10	0.20 ^a^ ± 0.20	10	0.20 ^a^ ± 0.20
		Melanomacrophage aggregate hyperplasia	2	0	0.00 ^a^ ± 0.00	30	0.60 ^a^ ± 0.30	30	0.60 ^a^ ± 0.30	20	0.40 ^a^ ± 0.26	20	0.40 ^a^ ± 0.26
**I_K_**			NA	NA	0.50 ^c^ ± 0.16	NA	10.50 ^a^ ± 2.54	NA	6.50 ^ab^ ± 1.57	NA	2.60 ^bc^ ± 0.85	NA	2.80 ^bc^ ± 0.97
**Tot-I**			NA	NA	1.00 ^c^ ± 0.33	NA	25.90 ^a^ ± 3.76	NA	14.50 ^b^ ± 3.57	NA	7.80 ^bc^ ± 1.90	NA	7.80 ^bc^ ± 1.75

Values are represented as the mean ± SE, *n* = 3 replicates (3 fish/replicate). The means within the same row carrying different superscripts (^a–c^) are significant at *p* < 0.05. IF, important factor; FQ, frequency I_L_, liver index; I_K_, kidney index; Tot-I, total index. NA; not applicable. G1, negative control, was fed a basal diet. G2 was exposed to MET 20.39 µg/L and fed a basal diet without PSM. G3, was exposed to MET 20.39 µg/L and fed a basal diet with 0.5% of PSM. G4, was exposed to MET 20.39 µg/L and fed a basal diet with 1.0% of PSM. G5 was exposed to MET 20.39 µg/L and fed a basal diet with 2.0% of PSM.

**Table 7:** Mortality rate, relative percent survival, clinical signs, and postmortem findings observed in survived *O. niloticus* fish in different experimental groups challenged with *Pseudomonas aeruginosa*.

	Experimental Groups					
		G1	G2	G3	G4	G5
**No. of challenged fish**	Number	15	15	15	15	15
**No. of survived fish**	Number	10	6	8	11	10
**Survival rate**	%	66.66	40	53.33	73.33	66.66
**Mortality rate**	%	33.33	60	46.66	26.66	33.33
**Relative percent survival**	%	0	−80	−39	20	0
**Abnormal swimming activity**	Number	4/10	4/6	4/8	2/11	4/10
	Score	++	+++	++	+	++
**Loss of appetite**	Number	6/10	5/6	5/8	4/11	5/10
	Score	+++	++++	+++	++	++
**Loss of reflexes**	Number	4/10	4/6	4/8	4/11	4/10
	Score	++	+++	++	++	++
**External skin lesion**	Number	7/10	5/6	5/8	5/11	6/10
	Score	+++	++++	+++	++	+++
**Postmortem change**	Number	7/10	6/6	5/8	5/11	6/10
	Score	+++	++++	+++	++	+++

The score of symptoms were recorded as follows: (+) weak; (++) mild; (+++) moderate; (++++) severe. G1, negative control, was fed a basal diet. G2 was exposed to MET 20.39 µg/L and fed a basal diet without PSM. G3 was exposed to MET 20.39 µg/L and fed a basal diet with 0.5% of PSM. G4 was exposed to MET 20.39 µg/L and fed a basal diet with 1.0% of PSM. G5 was exposed to MET 20.39 µg/L and fed a basal diet with 2.0% of PSM.

## Data Availability

Data is contained within the article.
